# Impact of deep brain stimulation on urogenital function in Parkinson’s disease: a systematic review and meta-analysis

**DOI:** 10.3389/fneur.2024.1397344

**Published:** 2024-07-04

**Authors:** Long Gao, Meng Wang, Mengdi Zhou, Wenjuan Yin, Xiaoming Cao

**Affiliations:** ^1^Department of Urology, First Hospital of Shanxi Medical University, Taiyuan, China; ^2^School of Basic Medical Sciences, Shanxi Medical University, Taiyuan, China

**Keywords:** systematic review, deep brain stimulation, Parkinson’s disease, urogenital function, lower urinary tract symptoms, sexual function

## Abstract

**Objectives:**

Deep Brain Stimulation (DBS) effectively treats Parkinson’s motor symptoms, but its effects on the urogenital system are debated.

**Methods:**

A research was conducted in PubMed, Embase, Cochrane Library, Web of Science, and Scopus until February 27, 2024. We primarily focused on DBS’s impact on Parkinson’s patients’ Urine storage function, voiding function, sexual function, and quality of life.

**Results:**

Our meta-analysis included 14 studies. The main results showed that DBS resulted in fewer instances of urinary urgency (OR = 1.85, 95% CI: 1.26 to 2.70, *p* = 0.002) and increased maximum bladder capacity (MD = −66.10, 95% CI: −119.37 to −12.82, *p* = 0.02) in terms of urinary storage function. However, there were no significant differences in first desire to void and strong desire to void. In terms of voiding function, DBS showed significant improvements in maximum flow rate (MD = −0.64, 95% CI: −1.23 to −0.05, *p* = 0.03), post-void residual (MD = −6.79, 95% CI: 4.54 to 9.05, *P* < 0.00001) and detrusor pressure during maximum flow (MD = −1.37, 95% CI: −2.73 to −0.02, *p* = 0.05). Additionally, there was no significant difference in sexual function between the two groups (MD = −1.41, 95% CI: −12.40 to 9.57, *p* = 0.80).

**Conclusion:**

DBS has demonstrated a certain degree of efficacy in ameliorating urinary storage and voiding function in patients with Parkinson’s disease. However, certain urodynamic parameters or scores do not demonstrate any statistically significant disparities. Furthermore, DBS has no significant impact on erectile function in male Parkinson’s patients.

**Systematic review registration:**

https://www.crd.york.ac.uk/prospero/display_record.php?ID=CRD42023476661, identifier CRD42023476661.

## 1 Introduction

Due to the pronounced advantages of deep brain stimulation (DBS), including safety, reversibility, customizable adaptability, and technological maturity, it has progressively emerged as the preferred modality for surgical intervention. DBS, involving the implantation of electrodes in sites such as the subthalamic nucleus (STN), globus pallidus internus (GPi), or ventral intermediate nucleus (VIM), coupled with electrical stimulation, serves to ameliorate both motor and non-motor symptoms in Parkinson’s patients. In the past, the assessment of the therapeutic efficacy of DBS in Parkinson’s patients primarily centered on the domain of the motor system. However, in recent years, investigators have shifted their focus to the impact of DBS on non-motor symptoms in Parkinson’s patients. Research findings indicate that DBS can ameliorate symptoms of depression and anxiety (1), enhance sleep quality (2), and modulate gastrointestinal motility (3). Furthermore, urinary function and reproductive function are also crucial aspects of non-motor symptoms. A study incorporating 1,072 Parkinson’s patients (with 60% being male and an average age of 67 years) revealed that nearly 60% of these patients exhibited urogenital system symptoms, with approximately 20% experiencing sexual dysfunction (4). Notably, the presence of urogenital non-motor symptoms was significantly correlated with a more prolonged disease course compared to patients without such symptoms (4).

Nevertheless, pertinent research concerning the impact of DBS on the urogenital system of Parkinson’s disease (PD) patients is notably sparse and marked by contradictory findings. Currently, there exists only one meta-analysis (5) that has examined the influence of DBS on urinary dynamic parameters in individuals with PD.

Therefore, we opt to conduct a comprehensive analysis of existing relevant research outcomes. On one hand, we will delve into the impact of DBS on Lower urinary tract symptoms (LUTS) by scrutinizing clinical symptoms describing storage and voiding phases, urodynamic parameters, and pertinent scale scores in PD patients. On the other hand, we will explore the effects of DBS on the sexual function of Parkinson’s patients. The study aims to furnish clinicians with a more comprehensive comprehension regarding the holistic impact of DBS on the urinary system of patients with PD. This endeavor will facilitate their articulation of the surgical benefits to patients, particularly when confronted with those afflicted by debilitating LUTS.

## 2 Methods

### 2.1 Search strategy

This meta-analysis adheres to the guiding principles of The Preferred Reporting Items for Systematic Reviews and Meta-Analyses (PRISMA) (6). The review protocol has been registered with PROSPERO (registration number: CRD42023476661), and detailed information is available on the PROSPERO website^1^. Two reviewers (Long Gao and Meng Wang) independently conducted searches in databases including PubMed, Embase, Cochrane Library, Web of Science, and Scopus. The search was conducted up to February 27, 2024, encompassing all literature since the inception of the databases. A combined strategy of subject terms and free-text terms was employed, with specific search terms including “parkinson disease,” “deep brain stimulation,” and (“lower urinary tract symptom” or “genital system”). Additionally, a secondary search of references in the included literature was conducted for supplementation. An example of searches in Embase are provided in Supplementary material 1. It is noteworthy that this study imposes no language restrictions.

### 2.2 Inclusion and exclusion criteria

Inclusion Criteria: (1) Study types include randomized controlled trials, non-randomized controlled studies, case–control studies, and cohort studies. (2) Study participants are clinically diagnosed with PD. (3) Regarding intervention measures, the experimental group undergoes DBS, while the control group does not undergo DBS surgery or electrode implantation without electrical stimulation. (4) At least one of the following study outcomes is included: Urinary urgency, First desire to void (FDV), Strong desire to void (SDV), Maximum bladder capacity (MBC), Maximum flow rate (MFR), Detrusor pressure during maximum flow (DPMF), Post void residual (PVR), Overactive Bladder Symptoms Score (OABSS), American Urological Association Symptom Index (AUA-SI) and International Index of Erectile Function (IIEF). Exclusion Criteria: (1) Incomplete or missing data for outcome indicators. (2) Literature types include individual case reports, reviews, letters, conference abstracts, experiential summaries, or books. (3) Articles that, despite attempts to contact the authors, cannot be accessed for the full text and complete data.

### 2.3 Selection process and data abstraction

All identified literature was imported into bibliographic management software (EndNote X9; Thomson Reuters, Philadelphia, PA, USA) and categorized through drag-and-drop functionality. Two reviewers (Mengdi Zhou and Meng Wang) independently examined the titles and abstracts of the literature, excluding those evidently unrelated to the topic. Subsequently, full texts were read, and literature was filtered according to predetermined criteria. Discrepancies were resolved through consultation between the two reviewers, and if consensus was not reached, a third author (Long Gao) was consulted. We utilized a pre-designed data extraction table to extract the required information. Baseline data encompassed: (Year, Country, Study type, Total patients, Mean age, Mean duration of neurological disorder, Site of stimulation). Outcome indicators comprised: Urinary urgency, FDV, SDV, MBC, MFR, DPMF, PVR, OABSS, AUA-SI, and IIEF. Additionally, we incidentally collected the reported Quality of Life (QoL) scores from the included literature.

### 2.4 Literature quality and risk of bias assessment

Two researchers conducted a quality assessment of the included literature. In cases of discrepancies during the quality assessment, resolution was achieved through consultation with a third-party researcher. In this meta-analysis, literature other than Pedro2020 consisted of self-controlled studies, whereas Pedro2020 was categorized as a cohort study. For cohort studies (RC: Retrospective cohort), we employed the Newcastle-Ottawa Scale (NOS) (7 for quality assessment). For non-randomized controlled trials (non-RCT), the Methodological Index for Non-Randomized Studies (MINORS) (8) was used for quality evaluation. The Newcastle-Ottawa Scale rates the quality of literature on a scale of 9 stars, with a score of ≥6 stars considered as high-quality literature. For non-randomized controlled trial methodological assessment, the MINORS is applicable to non-randomized controlled trials. Studies with a control group can achieve a maximum score of 24, while studies without a control group can score a maximum of 16. Higher scores indicate higher methodological quality and lower risk of bias. These assessment criteria contribute to ensuring that the literature included in the analysis maintains high quality and reliability.

### 2.5 Statistical analysis

The data from the included studies were imported into Review Manager software (The Cochrane Collaboration, RevMan version 5.4) for a comprehensive meta-analysis. The significance level was set at *p* < 0.05. For continuous variables, the mean difference (MD) and its 95% confidence interval (CI) were estimated as the effect size. For binary variables, the effect size was estimated using the odds ratio (OR) and its 95% CI. Heterogeneity was assessed using the *I*^2^ test, if I^2^is greater than 50%, the heterogeneity is deemed substantial, and the random-effects model should be employed. If *I*^2^ is less than 50%, it suggests that the heterogeneity is acceptable, and a fixed-effect model should be utilized. For the group with high heterogeneity, we systematically excluded individual literature articles using Stata software (StataCorp, College Station, TX, USA) in order to conduct a sensitivity analysis. Subsequently, sensitivity analysis forest plots were generated to visually represent the results and account for potential biases caused by the high heterogeneity. If sensitivity analysis indicates instability in the results, we will further conduct subgroup analyses to investigate the potential reasons behind it. Moreover, considering the potential interference of dopaminergic agents (such as levodopa or dopamine agonists, MAOIs, etc.) or commonly used urological medications (such as alpha-adrenergic blockers) on post-DBS urinary function in PD patients, we will stratify the groups based on whether these medications were controlled for in the study, and subsequently conduct subgroup analyses to ascertain whether our conclusions are influenced by the use of these two classes of medications. Finally, quantitative assessment of publication bias was conducted using Egger’s test. A *p*-value ≥0.05 indicates no apparent publication bias, while a *p*-value <0.05 suggests the presence of publication bias, warranting further investigation.

## 3 Results

### 3.1 Literature retrieval results and basic characteristics

**Figure 1:**
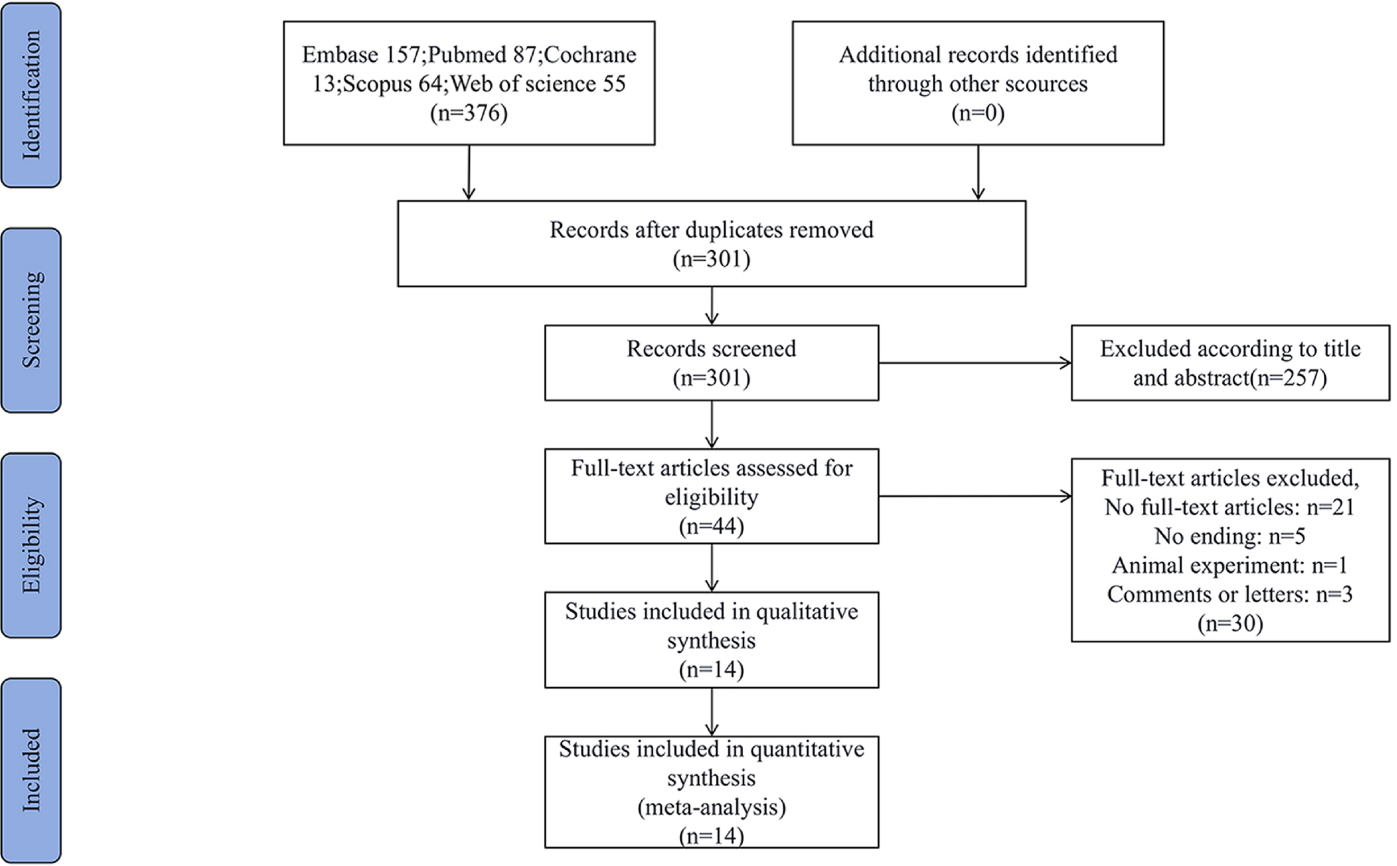
Literature search and selection.

After a comprehensive and systematic search, a total of 376 articles were identified. Following the removal of duplicate publications, two researchers independently screened the articles based on the inclusion and exclusion criteria mentioned earlier. Ultimately, 14 eligible articles (9–16) were included, consisting of 1 retrospective cohort study (13) and 13 self-controlled studies (9–12, 14–16). The literature screening process is illustrated in Figure 1, and the basic characteristics of the included studies, along with the results of the quality assessment, are presented in Table 1.

**Table 1:** Literature basic information and literature quality evaluation results.

Study	Year	Country	Study type	Total patients [male/female]	Mean age [years]	Mean duration of neurological disorder [years]	Site of stimulation	Outcomes	MINORS/NOS
Finazzi-Agrò (17)	2003	Italy	Non-RCT	5[3/2]	63	15	STN	MBC; DPMF; MFR	10
Seif (18)	2004	Germany	Non-RCT	16[7/9]	62	15	STN	MBC; FDV; MFR; PVR	9
Herzog (19)	2006	Germany	Non-RCT	11[5/6]	57.7	15.2	STN	FDV; SDV;	11
Shimizu (20)	2007	Japan	Non-RCT	6[4/2]	65.8	NR	STN	MBC; MFR; FVR	11
Herzog (21)	2008	Germany	Non-RCT	9[5/4]	60	15.2	STN	FDV; SDV;	11
Mock (22)	2016	US	Non-RCT	33[24/9]	62.3	7.8	STN/GPi	AUA-SI; QoL	12
Roy (9)	2018	UK	Non-RCT	5[5/0]	62.8	22.4	PPN	MBC	10
Kurcova (10)	2018	Czech Republic	Non-RCT	24[20/4]	62	8	STN	IIEF	11
Yamamoto (11)	2018	Japan	Non-RCT	28[NR]	65.5	11.8	STN/GPi	OABSS; FDV; SDV; MFR; PVR	9
Zong (12)	2019	China	Non-RCT	220[160/60]	61.3	7.7	NR	OABSS; AUA-SI; MBC; DPMF; MFR; PVR; QoL; Urinary urgency	10
Pedro (13)	2020	Portugal	RC	With DBS-21[12/9] vs. Without DBS-19[9/10]	With DBS-62.9 vs. Without DBS-60.7	With DBS-17.9 vs. Without DBS-10.4	STN	IIEF	7
Liang (14)	2021	China	Non-RCT	20[0/20]	60.6	NR	STN	OABSS; QoL	10
Sartori (15)	2022	Switzerland	Non-RCT	39[27/12]	63	13.8	STN/GPi/VIM	MBC; DPMF; FDV; SDV; MFR; PVR	11
Wolz (16)	2012	Germany	Non-RCT	34[NR]	NR	NR	STN	Urinary urgency	12

MINORS, Methodological Index for Non-Randomized Studies; NOS, Newcastle-Ottawa Scale for cohort studies; STN, subthalamic nucleus; GPi, globus pallidus internus; PPN, pedunculopontine nucleus; VIM, ventral intermediate nucleus of the thalamus; non-RCT, non-Randomized Control Trial; RC, Retrospective cohort; NA, not applicable; NR, not reported; OABSS, Overactive Bladder Symptoms Score; AUA-SI, American Urological Association Symptom Index; MBC, Maximum bladder capacity; DPMF, Detrusor pressure during maximum flow; FDV, First desire to void; SDV, Strong desire to void; MFR, Maximum flow rate; PVR, Post-void residual; SHIM, Sexual Health Inventory for Men; IIEF, International Index of Erectile Function; QoL, Quality of Life.

### 3.2 Methodological quality assessment

Following quality assessment, the non-randomized controlled trials included in this meta-analysis achieved MINORS scores consistently exceeding 9 points. The sole cohort study received a score of 7 on the NOS scale, classifying it as a high-quality study (Table 1).

### 3.3 Urinary storage function

**Figure 2:**
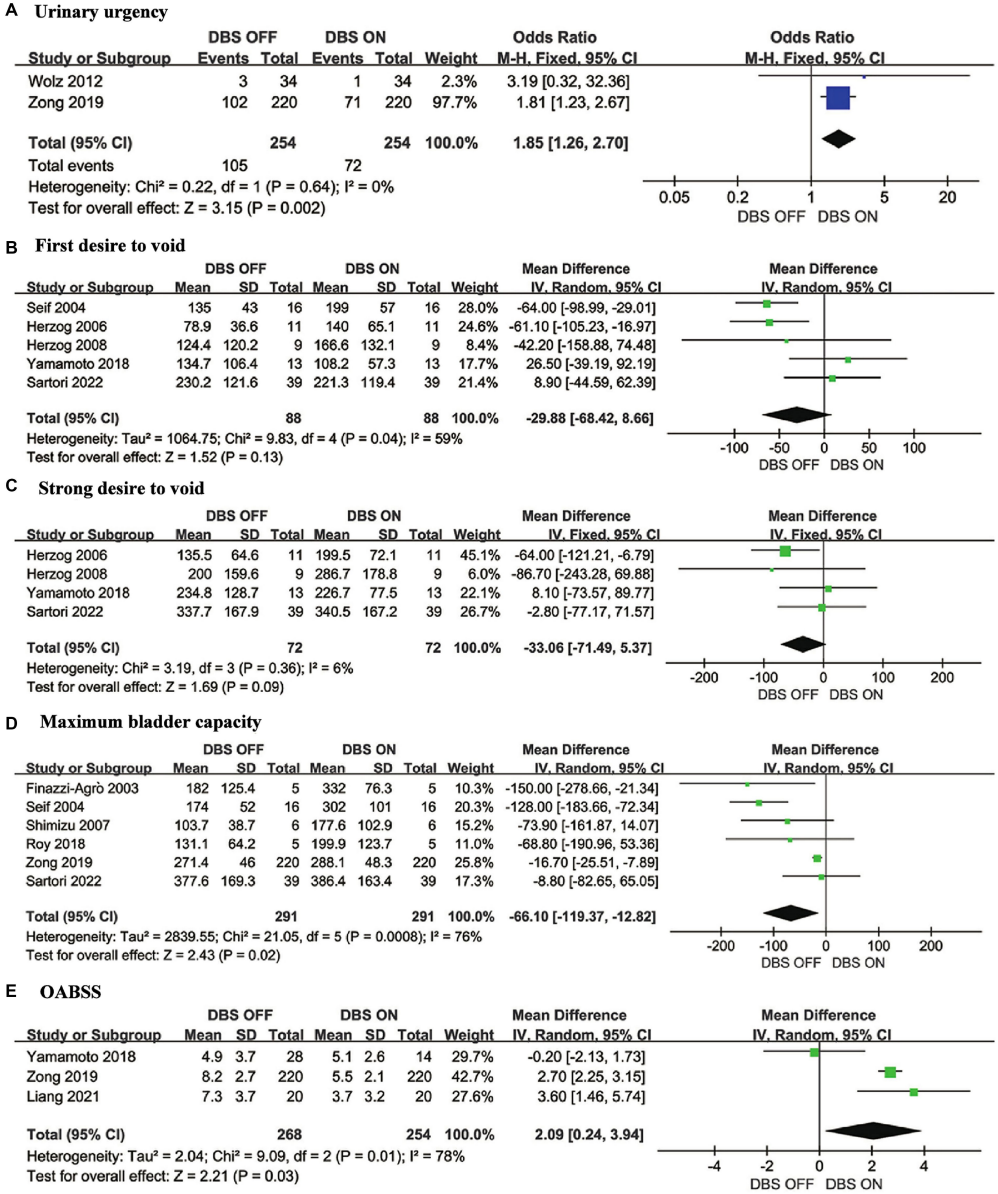
Forest plots for urinary storage function. **(A)** Urinary urgency, **(B)** First desire to void, **(C)** Strong desire to void, **(D)** Maximum bladder capacity and **(E)** OABSS.

Urinary urgency, FDV, SDV, MBC, and OABSS were the outcomes representing storage phase function, and we conducted a meta-analysis on these parameters. As a typical clinical manifestation among storage phase symptoms, urinary urgency exhibited a significant improvement under DBS ON conditions (OR = 1.85, 95% CI: 1.26 to 2.70, *p* = 0.002) (Figure 2A). For urodynamic parameters, smaller values of FDV, SDV, and MBC indicate more severe storage phase disorders in patients (23, 24). Among them, FDV (MD = −29.88, 95% CI: −68.42 to 8.66, *p* = 0.13) (Figure 2B), SDV (MD = −33.06, 95% CI: −71.49 to 5.37, *p* = 0.09) (Figure 2C), and MBC (MD = −66.10, 95% CI: −119.37 to −12.82, *p* = 0.02) (Figure 2D). As a scale assessing the severity of storage phase symptoms, higher OABSS indicate more severe over active bladder (OAB) symptoms in patients (11). The meta-analysis results show that DBS ON conditions significantly reduce OABSS in Parkinson’s patients compared to DBS OFF conditions (MD = 2.09, 95% CI: 0.24 to 3.94, *p* = 0.03) (Figure 2E).

### 3.4 Urinary voiding function

**Figure 3:**
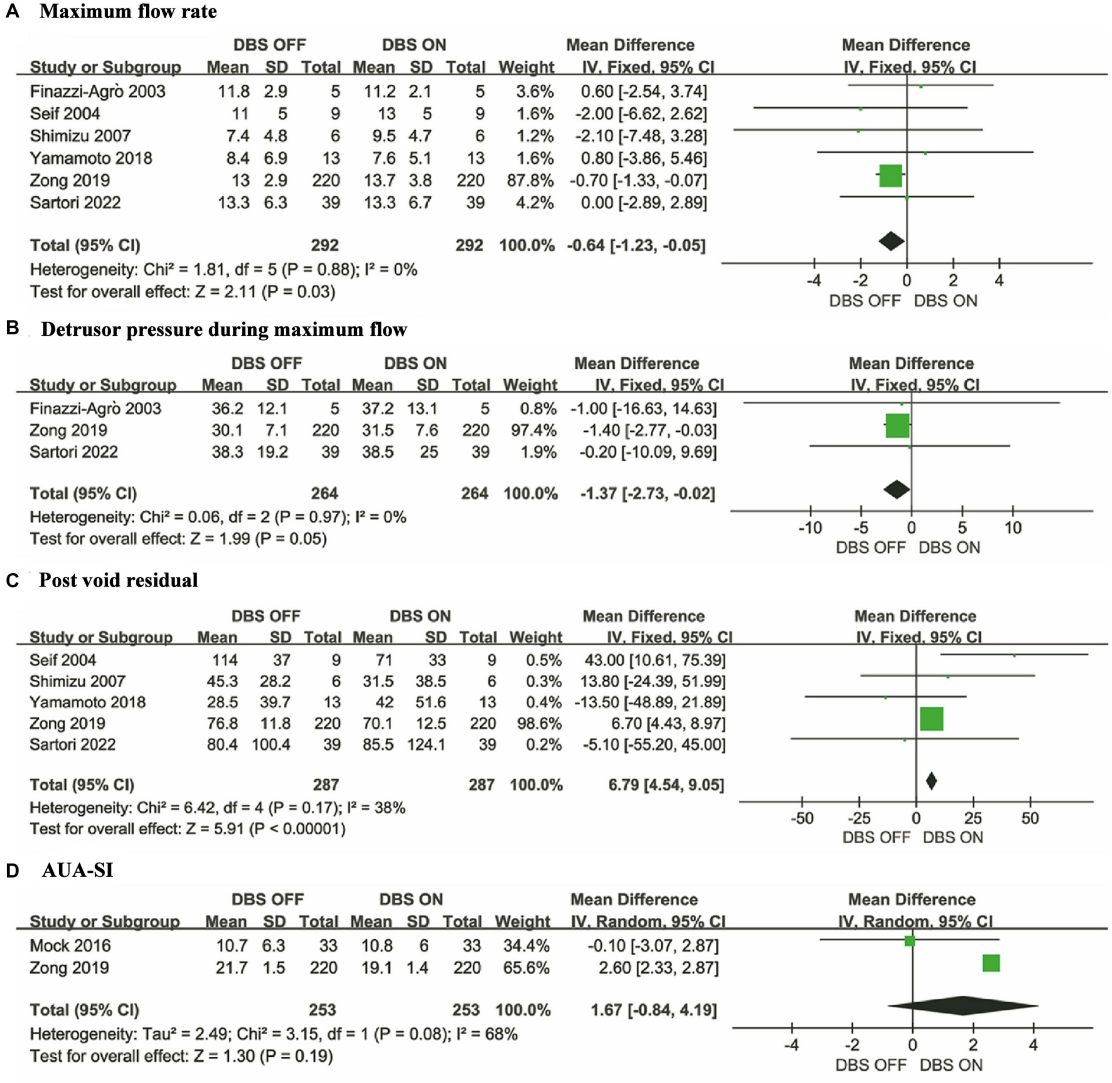
Forest plots for urinary voiding function. **(A)** Maximum flow rate, **(B)** Detrusor pressure during maximum flow, **(C)** Post void residual and **(D)** AUA-SI.

We conducted a meta-analysis on MFR, DPMF, PVR, and AUA-SI, which represent voiding phase function. Smaller MFR and DPMF correspond to more severe voiding difficulties (12). The meta-analysis results indicate that, compared to DBS OFF conditions, both MFR (MD = −0.64, 95% CI: −1.23 to −0.05, *p* = 0.03) (Figure 3A) and DPMF (MD = −1.37, 95% CI: −2.73 to −0.02, *p* = 0.05) (Figure 3B) significantly increased under DBS ON conditions. Specifically, larger PVR values are associated with more severe storage and voiding phase symptoms (25). Five studies (11, 12, 15, 18, 20) reported the difference in PVR between DBS OFF and DBS ON conditions, and the results showed no statistical difference in PVR between the two groups (MD = 6.79, 95% CI: 4.54 to 9.05, *P* < 0.00001) (Figure 3C). Higher AUA-SI indicate more severe obstructive symptoms (22). The meta-analysis reveals no significant difference in AUA-SI between DBS ON and DBS OFF conditions (MD = 1.67, 95% CI: −0.84 to 4.19, *p* = 0.19) (Figure 3D).

### 3.5 Sexual function

**Figure 4:**
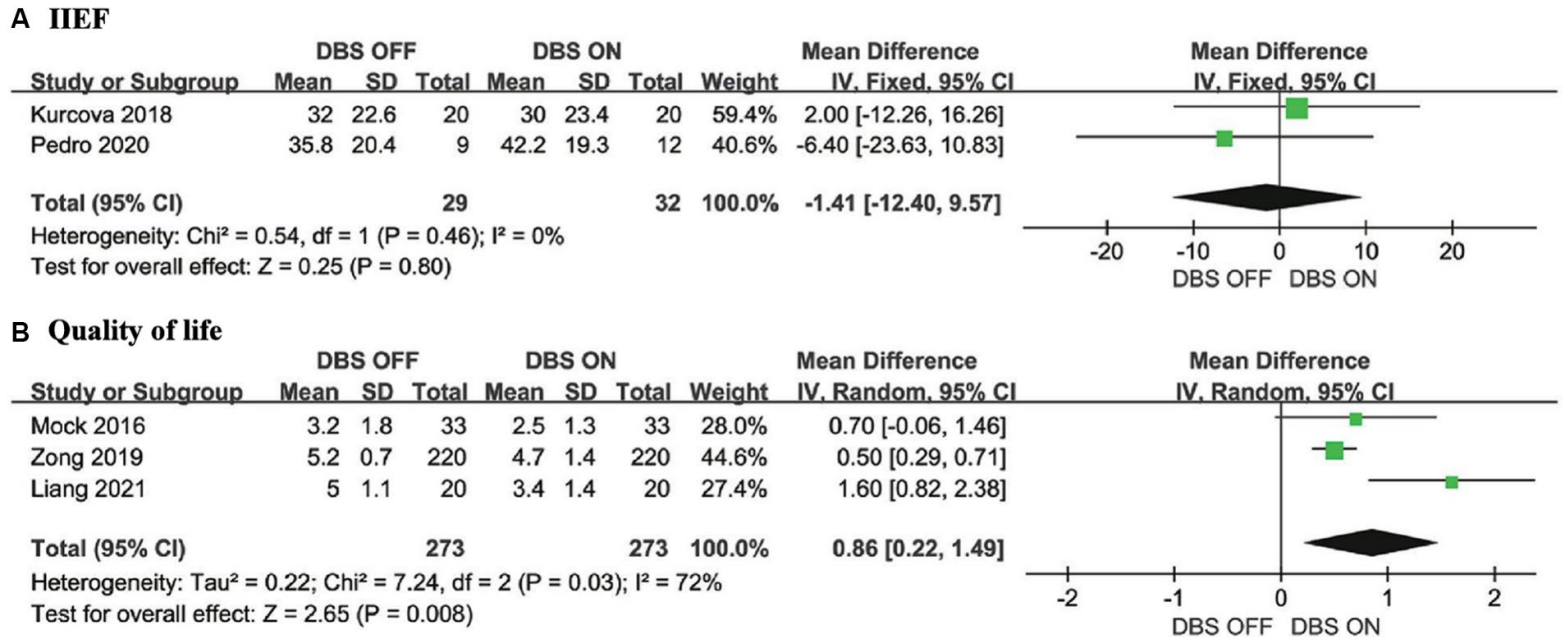
**(A)** Forest plots for IIEF and **(B)** Forest plots for Quality of life.

IIEF is a comprehensive scale that reflects male erectile function, orgasmic function, sexual desire, satisfaction with intercourse, and overall satisfaction. Higher scores indicate better male sexual function (13). Our meta-analysis indicates that there is no significant improvement in IIEF when comparing DBS ON to DBS OFF conditions (MD = −1.41, 95% CI: −12.40 to 9.57, *p* = 0.80) (Figure 4A).

### 3.6 Quality of life

Smaller QoL scores imply better quality of life (22). Three studies reported the impact of DBS on the scores of PD patients. Our meta-analysis indicates a significant decrease in QoL scores for PD patients under DBS ON conditions compared to DBS OFF conditions (MD = 0.86, 95% CI: 0.22 to 1.49, *p* = 0.008) (Figure 4B).

### 3.7 Sensitivity analysis

**Figure 5:**
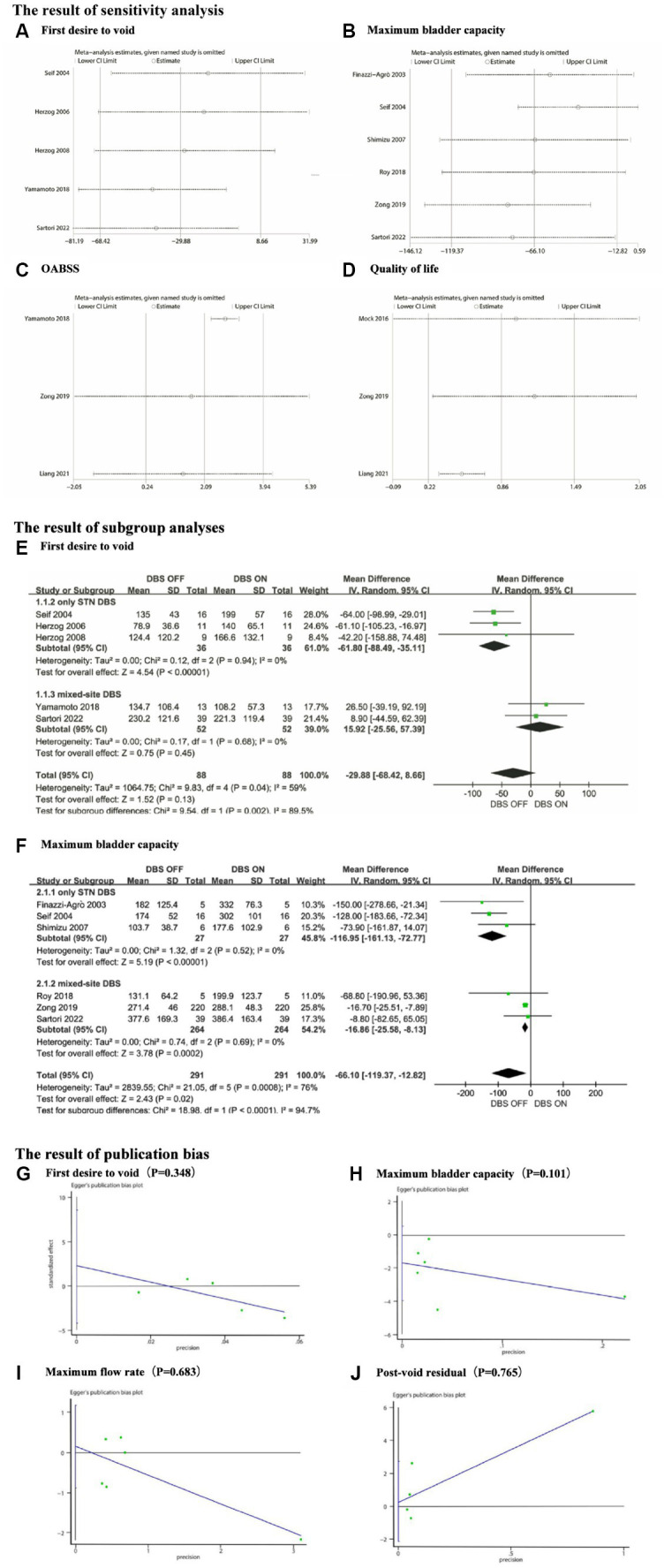
**(A)** Sensitivity analysis for First desire to void, **(B)** Sensitivity analysis for Maximum bladder capacity, **(C)** Sensitivity analysis for OABSS, **(D)** Sensitivity analysis for Quality of life, **(E)** The subgroup analyses for First desire to void, **(F)** The subgroup analyses for Maximum bladder capacity, **(G)** The publication bias for First desire to void, **(H)** The publication bias for Maximum bladder capacity, **(I)** The publication bias for Maximum flow rate and **(J)** The publication bias for Post-void residual.

In the meta-analysis of FDV, MBC, OABSS, AUA-SI, and QoL scores, we observed significant heterogeneity, with I^2^ values of 59, 76, 78, 68, and 72%, respectively. Sensitivity analysis was not performed for AUA-SI as only two articles were included in this group. We conducted sensitivity analysis using Stata software for the remaining parameters and generated forest plots after excluding each study one by one (Figures 5A–D). The analysis results indicate: For the FDV group, the outcome significantly changed after the exclusion of Sartori 2022 (15) and Yamamoto 2018 (11). The exclusion of Yamamoto 2018 led to a decrease in *I*^2^ from 59 to 45%. In the MBC group, the outcome significantly changed after the exclusion of Roy 2018 (9), Finazzi-Agrò 2003 (17), and Seif 2004 (18). The exclusion of Seif 2004 (18) led to a decrease in *I*^2^ from 79 to 29%. For the OABSS group, the outcome significantly changed after the exclusion of Zong 2019 (12) and Liang 2021 (14). The exclusion of Yamamoto 2018 led to a decrease in *I*^2^ from 78 to 0%. In the QoL scores group, the outcome significantly changed after the exclusion of Mock 2016 (22). The exclusion of Liang 2021 (14) led to a decrease in I^2^from 72 to 0%. In summary, the outcomes for FDV, MBC, OABSS, AUA-SI, and QoL scores are all unstable.

### 3.8 Subgroup analysis

To further explore the heterogeneity sources, we conducted subgroup analyses on two outcome measures, FDV and MBC. According to the stimulation site, we divided the participants into two subgroups: those who received stimulation solely in the STN (only STN DBS), and those who received stimulation involving other brain regions in addition to the STN (mixed-site DBS). As shown in Figures 5E,F, both the only STN DBS subgroup and the mixed-site DBS subgroup exhibited markedly low within-group heterogeneity in both the FDV and MBC cohorts. This suggests that the stimulation site may be one of the contributing factors to the observed heterogeneity in terms of FDV and MBC. Due to the limited number of included studies, we were unable to conduct subgroup analyses on the OABSS group and Quality of Life group. Furthermore, we conducted subgroup analyses regarding the interference of dopaminergic agents on the study outcomes. The findings indicated incongruity in the direction of effect sizes across four parameters (FDV, SDV, MFR, and PVR) between the group with balanced dopaminergic agents and the overall people. In other words, upon mitigating inter-group differences in dopaminergic agents, a reversal in the direction of effect sizes was observed. Unfortunately, due to insufficiently detailed data, we were unable to further ascertain whether this influence was favorable or adverse. Additionally, we did not undertake subgroup analyses for commonly used urological medications. For a comprehensive analysis, refer to Supplementary material 2.

### 3.9 Publication bias

We performed a quantitative evaluation of publication bias using Egger’s test for FDV, MBC, MFR, and PVR. The results revealed no significant publication bias for any of these groups. The corresponding Egger plots and *p*-values are presented in Figures 5G–J.

## 4 Discussion

### 4.1 Lower urinary tract symptoms

Lower Urinary Tract Symptoms are commonly observed in patients with PD, encompassing both storage symptoms (overactive bladder symptoms or OAB) and voiding symptoms (obstruction symptoms). These symptoms may manifest independently or coexist in individuals with PD (26, 27).

#### 4.1.1 Urinary storage function

Nocturia is one of the most commonly reported non-motor symptoms in patients with PD (28). In this context, key factors contributing to the reduction in functional bladder capacity include decreased compliance of the bladder wall (resulting in decreased MBC), overactivity of the detrusor muscle (reflected in OABSS), and excessively large PVR (28). On the other hand, patients with PD often experience a significant increase in nocturnal urine production (29). The combined impact of these two aspects contributes to the manifestation of nocturia. Furthermore, the reduction in functional bladder capacity is also a significant factor contributing to daytime frequency and urgency (30, 31). However, it should be noted that if DBS excessively reduces detrusor muscle activity, it may also lead to urinary retention (32).

Unfortunately, due to the limited number of included articles, we were only able to conduct a meta-analysis for the symptom of Urinary urgency. The results show that DBS significantly improves Urinary urgency in patients with PD (*p* < 0.05). In clinical practice, physicians often utilize the collection of urinary dynamic parameters to corroborate the LUTS in patients. Our meta-analysis results indicate a significant improvement in MBC, PVR and OABSS in Parkinson’s patients after receiving DBS treatment (*p* < 0.05).

The neurological mechanisms by which DBS improves storage symptoms in patients with PD have been a focus of researchers. One theory suggests that the loss of output from the basal ganglia reduces the inhibitory control of the cortical bladder reflex in Parkinson’s patients, leading to overactivity of the detrusor muscle and the foundation of storage symptomatology (31). Herzog et al.’s study (21) found that, in the STN-DBS ON state, neural activity in the thalamus and insular cortex was enhanced under the modulation of the PAG pathway, indicating that STN-DBS restores the basal ganglia circuitry to some extent. A recent experiment on a PD rodent model (33) further revealed this mechanism. In the STN-DBS ON state, when the PAG pathway was activated (i.e., generating a sense of urgency), STN-DBS may promote the normalization of abnormal alpha power in the medial prefrontal cortex (mPFC) by increasing the power of local field potentials, reducing levels of levodopa, dopamine, serotonin, and their metabolites in the mPFC. This inhibition of abnormal alpha power in the mPFC suppresses detrusor muscle activity, thereby reversing OAB symptoms caused by overactive detrusor muscle.

#### 4.1.2 Urinary voiding function

Obstruction symptoms in PD, while relatively uncommon compared to storage symptoms, can be equally troublesome, particularly for male individuals. The causes of obstruction symptoms in PD patients are diverse. With the sluggish movement of the sphincter muscles, some PD patients may experience Detrusor Overactivity (DO) during storage but present with detrusor areflexia during voiding. In terms of urodynamic parameters, this is reflected in reduced MFR and DPMF, alongside increased bladder capacity, FDV, SDV, and PVR. This combination is estimated to occur in up to 18% of PD patients (34). Our meta-analysis indicates a significant improvement in both MFR and DPMF after DBS (*p* ≤ 0.05). Regrettably, we have yet to identify any studies that elucidate the pathophysiological mechanisms by which DBS affects voiding symptoms in PD patients. However, it is possible that we may have overlooked relevant research.

Additionally, PD primarily affects the elderly population, with benign prostatic hyperplasia being common in elderly males, and bladder neck stenosis being more prevalent in elderly females. These age-related conditions are significant contributors to urinary system symptoms in some PD patients (35). As demonstrated by Zong et al.’s study, females exhibited greater improvement in AUA-SS, OAB-SS, and QoL scores after receiving DBS (12), which might be attributed to anatomical differences related to gender.

### 4.2 Sexual function

Up to 60% of male PD patients acknowledge the presence of erectile dysfunction, ejaculation issues, and difficulty reaching climax (36, 37). Simultaneously, as many as 75% of female PD patients report sexual problems such as vaginal dryness, decreased libido, and difficulty reaching climax (38). Castelli et al. conducted a groundbreaking study exploring the impact of DBS on the sexual function of PD patients. After DBS surgery, male PD patients experienced a slight but significant improvement in sexual function, particularly those under 60 years old, while there was no significant change in sexual satisfaction for females (39). A pioneering animal experiment on monkeys found that erectile performance is directly or indirectly related to STN/GPi (40). Additionally, literature reports indicate that patients after STN-DBS may exhibit heightened libido and impulsive tendencies (41, 42), possibly due to electrode impact on adjacent structures in the basal ganglia, which are associated with motivation, emotion, and cognitive functions (41). However, our meta-analysis results indicate no significant association between DBS and the IIEF in males.

### 4.3 Quality of life

The impact of non-motor symptoms (NMS), including LUTS, on the quality of life is no less significant than that of motor impairments, particularly in advanced stages of PD (43–45). Our meta-analysis reveals a substantial improvement in the QoL scores during DBS ON compared to DBS OFF, despite encountering notable heterogeneity (MD = 0.86, 95% CI: 0.22 to 1.49, *p* = 0.008). Considering evidence demonstrating improved QoL scores only in patients receiving STN-DBS compared to GPi-DBS (22), the observed heterogeneity within the QoL scores group in our meta-analysis may stem from variations in electrode stimulation sites. It needs to be clarified that there is indeed a substantial body of literature examining the impact of DBS on QoL scores in PD patients. This encompasses not only the three studies included in our analysis but also those studies that did not report urogenital parameters but did report QoL scores. However, we only evaluated the former. Similar to our findings, a wealth of studies not included in our analysis support the improvement of QoL with DBS. Furthermore, the QoL scores in the study by Mock et al. were derived solely from the last question of the AUA-SI questionnaire, reflecting only the QoL concerning urogenital symptoms, whereas the QoL in the other two studies reflected the overall QoL of each individual.

### 4.4 Strengths and limitations

This is the latest systematic review on the impact of DBS on the urinary and reproductive systems of patients with PD. In contrast to previous studies, we have collected and synthesized data on symptoms, urodynamic parameters, and subjective assessment scores, thus enriching the dimensions of evidence. We have also categorized the outcomes of interest according to storage and voiding functions, which will help neurosurgeons better conceptualize the impact of DBS on lower urinary tract function, rather than merely focusing on those tedious urological parameters. Additionally, research regarding the effects of DBS on sexual function in PD patients is scarce in this field, and there is a lack of relatively consistent outcome measures among studies, which may have led to a dearth of meta-analyses summarizing the effects of DBS on sexual function. Our work will encourage researchers to pay more attention to this relatively blank area and optimize their experimental designs in outcome assessments.

However, some limitations should be considered. Firstly, the current literature predominantly consists of non-randomized controlled before-after studies, lacking a sufficient number of high-quality randomized controlled trial or observational studies. We anticipate more high-quality research in this field in the future. Secondly, in the existing literature on the impact of DBS on LUTS in Parkinson’s patients, most studies focus on subthalamic nucleus (STN) stimulation or fail to specifically specify the stimulation site, neglecting differences between STN, globus pallidus internus (GPi), and ventral intermediate nucleus (VIM) stimulation. Due to the insufficient number of studies exploring the differential effects of stimulation sites, we were unable to conduct detailed subgroup analyses or meta-regressions. Thirdly, gender may be a potential factor influencing outcome measures due to anatomical differences in the urinary system. However, the scarcity of studies providing independent effect sizes for males and females hindered an in-depth gender analysis. Fourthly, due to the limited scope of the studies included, we were unable to exclude patients who had pre-existing urological conditions prior to undergoing DBS surgery. This may have introduced potential confounding variables impacting the study outcomes. Fifthly, our subgroup analysis corroborated that the utilization of dopaminergic agents indeed interfered with the outcomes on certain urodynamic parameters. We hope future researchers will pay more attention to potential confounding factors such as stimulation sites, gender and medications, delving into their impacts. Lastly, we observed substantial heterogeneity in the meta-analysis of the FDV/MBC/OABSS/QoL scores group, and sensitivity analysis indicated instability in the results. However, the underlying reasons for this heterogeneity remain elusive. Despite employing a random-effects model, caution is warranted in interpreting these results.

## 5 Conclusion

Our meta-analysis results show that there is a discernible correlation between DBS and urinary storage symptoms, including urinary urgency, maximum bladder capacity, and Overactive Bladder Symptom Score, as well as voiding symptoms such as post void residual, detrusor pressure during maximum flow and maximum flow rate. Noteworthy trends towards improvement were observed in first desire to void and strong desire to void for storage symptoms, and American Urological Association Symptom Index for voiding symptoms, although statistical significance eluded these trends. A limited number of studies tentatively suggest a potential absence of significant association between DBS and male erectile function. Nevertheless, given the scarcity of high-quality randomized controlled trials and the presence of significant heterogeneity, further scrutiny is warranted for the validation of these findings.

## Data availability statement

The original contributions presented in the study are included in the article/Supplementary material, further inquiries can be directed to the corresponding authors.

## Author contributions

LG: Writing – original draft, Conceptualization, Methodology. MW: Writing – original draft, Conceptualization, Methodology. MZ: Writing – original draft, Conceptualization, Methodology. WY: Writing – review & editing, Supervision. XC: Writing – review & editing, Funding acquisition.

## Footnotes

^1^ http://www.crd.york.ac.uk/PROSPERO
