# Supplementary material for: Impact of deep brain stimulation on urogenital function in Parkinson’s disease: a systematic review and meta-analysis
Source: Front Neurol. 2024 Jul 4;15:1397344. doi: 10.3389/fneur.2024.1397344 (PMC11254620; doi:10.3389/fneur.2024.1397344)
Supplement: Supplementary file 1 [file Table_1.DOCX]

# Supporting information 1. Example of search strategy

Databases: Embase, PubMed, Cochrane Central Register of Controlled Trials, Web of Science and Scopus

searched on 27 Feb 2024.

Example of Embase search on 27 Feb 2024:

.......................................................

No. Query Results Results Date

#14. #3 AND #6 AND #13 157 27 Feb 2024

#13. #9 OR #12 1,216,139 27 Feb 2024

#12. #10 OR #11 883,344 27 Feb 2024

#11. 'sexual intercourse frequency':ti,ab,kw OR 52,949 27 Feb 2024

'premature ejaculation':ti,ab,kw OR

impotence:ti,ab,kw OR iief:ti,ab,kw OR 'erectile

function':ti,ab,kw OR 'orgasmic

function':ti,ab,kw OR 'sexual desire':ti,ab,kw OR

'sexual dysfunction':ti,ab,kw

#10. 'genital system'/exp 839,732 27 Feb 2024

#9. #7 OR #8 367,927 27 Feb 2024

#8. 'lower urinary tract symptom':ti,ab,kw OR 'lower 361,405 27 Feb 2024

urinary tract function':ti,ab,kw OR 'urinary

dysfunction':ti,ab,kw OR 'urinary tract

function':ti,ab,kw OR bladder:ti,ab,kw OR

'intermittent catheterization':ti,ab,kw OR

incontinence:ti,ab,kw OR micturition:ti,ab,kw OR

'first desire void':ti,ab,kw OR 'strong desire

void':ti,ab,kw OR 'maximum bladder

capacity':ti,ab,kw OR 'post-void

residual':ti,ab,kw OR 'daytime

frequency':ti,ab,kw OR 'nighttime

frequency':ti,ab,kw OR 'detrusor

overactivity':ti,ab,kw OR 'detrusor pressure

maximum flow rate':ti,ab,kw OR 'maximum flow

rate':ti,ab,kw OR 'voided volume':ti,ab,kw OR

'postvoid residual':ti,ab,kw OR oab:ti,ab,kw OR

'overactive bladder':ti,ab,kw OR 'international

prostate symptom score':ti,ab,kw OR ipss:ti,ab,kw

OR 'american urological association symptom

index':ti,ab,kw OR 'aua si':ti,ab,kw

#7. 'lower urinary tract symptom'/exp 20,182 27 Feb 2024

#6. #4 OR #5 65,471 27 Feb 2024

#5. 'deep brain stimulation':ti,ab,kw OR 'deep brain 34,254 27 Feb 2024

stimulations':ti,ab,kw OR 'stimulation, deep

brain':ti,ab,kw OR 'stimulations, deep

brain':ti,ab,kw OR 'brain stimulation,

deep':ti,ab,kw OR 'electrical stimulation of the

brain':ti,ab,kw OR dbs:ti,ab,kw

#4. 'brain depth stimulation'/exp 54,628 27 Feb 2024

#3. #1 OR #2 202,232 27 Feb 2024

#2. 'idiopathic parkinsons disease':ti,ab,kw OR 2,313 27 Feb 2024

'lewybody parkinsons disease':ti,ab,kw OR

'parkinsons disease, idiopathic':ti,ab,kw OR

'parkinsons disease, lewybody':ti,ab,kw OR

'parkinson disease, idiopathic':ti,ab,kw OR

'parkinsons disease':ti,ab,kw OR 'idiopathic

parkinson disease':ti,ab,kw OR 'lewy body

parkinson disease':ti,ab,kw OR 'primary

parkinsonism':ti,ab,kw OR 'parkinsonism,

primary':ti,ab,kw OR 'paralysis agitans':ti,ab,kw

#1. 'parkinson disease'/exp 201,906 27 Feb 2024
