# Supplementary material for: Impact of deep brain stimulation on urogenital function in Parkinson’s disease: a systematic review and meta-analysis
Source: Front Neurol. 2024 Jul 4;15:1397344. doi: 10.3389/fneur.2024.1397344 (PMC11254620; doi:10.3389/fneur.2024.1397344)
Supplement: Supplementary file 2 [file Data_Sheet_1.PDF]

Supplementary Material

1. Supplementary Tables

Supplementary Table1. Summary table of the usage of dopaminergic agents and commonly used urological medications.

Study	dopaminergic agents		Medications for urological/sexual function	
	Intervention was implemented 12 hours post cessation of medication/with no significant intergroup differences in drug effect	The confounding factor was not balanced/unreported medication status	Intervention was implemented 12 hours post cessation of medication/with no significant intergroup differences in drug effect	The confounding factor was not balanced/unreported medication status
Finazzi-Agrò 2003		√		√
Seif 2004	√			√
Herzog 2006	√			√
Shimizu 2007	√			√
Herzog 2008	√			√
Mock 2016	√			√
Roy 2018	√			√
Kurcova 2018		√		√
Yamamoto 2018		√		√
Zong 2019		√		√
Pedro 2020	√		√	
Liang 2021		√		√

Sartori 2022		√		√
Wolz 2012	√			√

2. Supplementary Figures

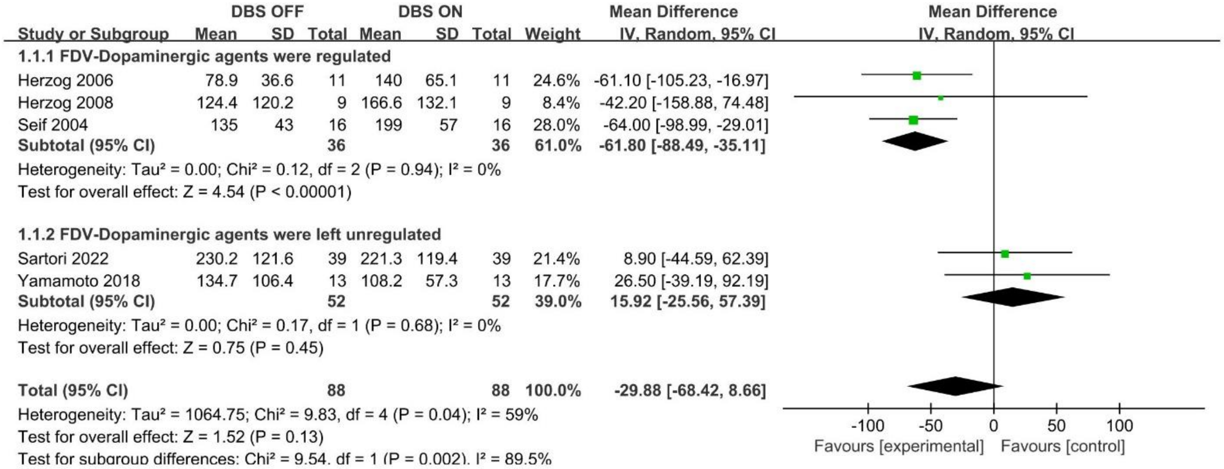

Supplementary Figure 1. Subgroup analysis of the impact of dopaminergic agents on First desire to void (FDV).

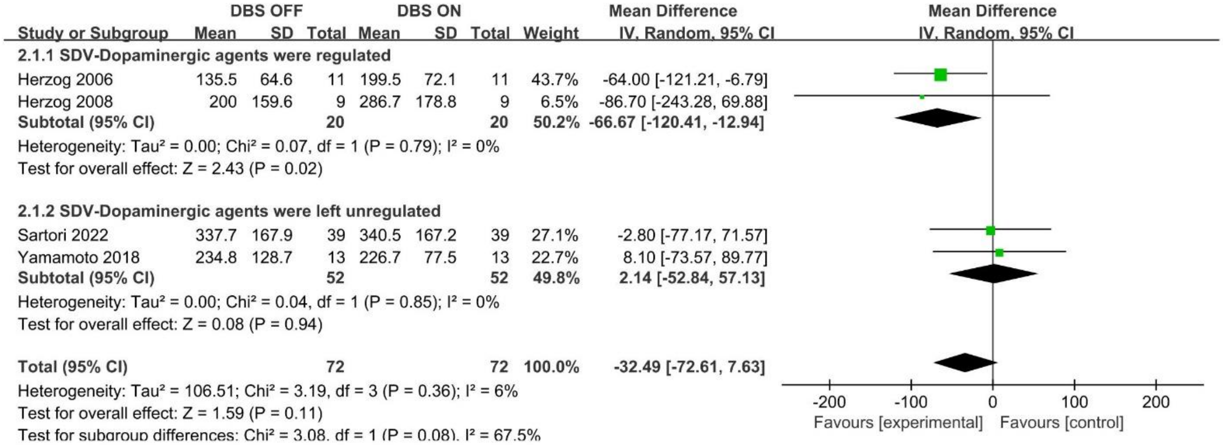

Supplementary Figure 2. Subgroup analysis of the impact of dopaminergic agents on Strong desire to void (SDV).

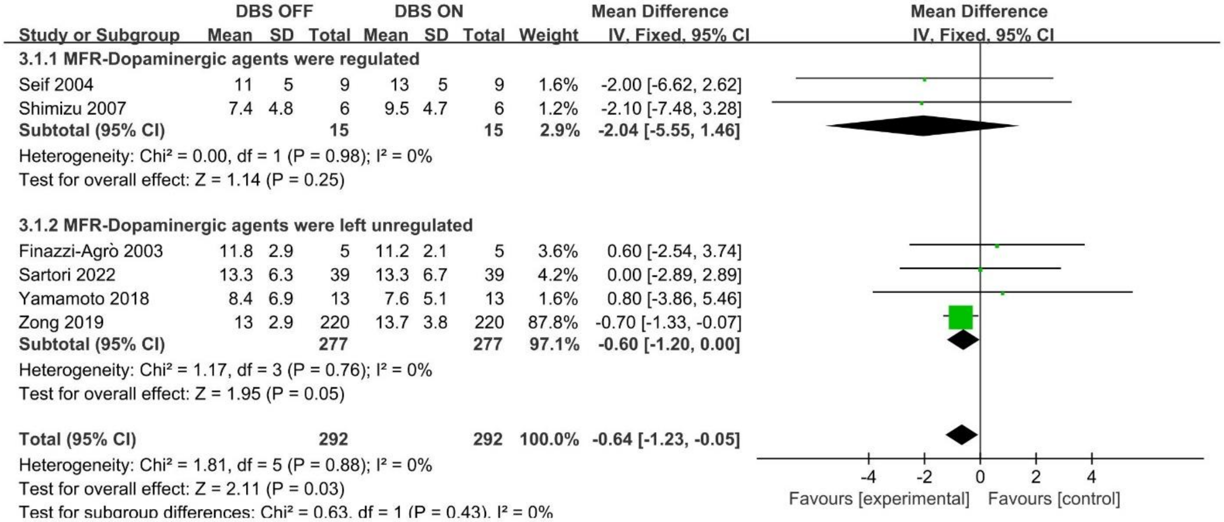

Supplementary Figure 3. Subgroup analysis of the impact of dopaminergic agents on Maximum flow rate (MFR).

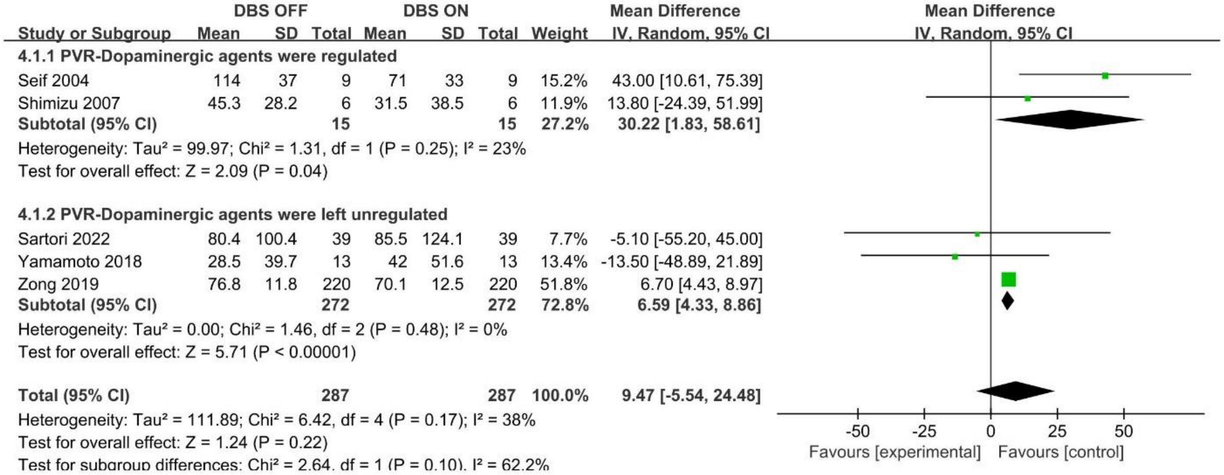

Supplementary Figure 4. Subgroup analysis of the impact of dopaminergic agents on Post-void residual (PVR).

Study or Subgroup	DBS OFF Mean	DBS OFF SD	DBS OFF Total	DBS ON Mean	DBS ON SD	DBS ON Total	Weight	Mean Difference IV, Random, 95% CI
**5.1.1 MBC-Dopaminergic agents were regulated**								
Roy 2018	131.1	64.2	5	199.9	123.7	5	11.0%	-68.80 [-190.96, 53.36]
Seif 2004	174	52	16	302	101	16	20.3%	-128.00 [-183.66, -72.34]
Shimizu 2007	103.7	38.7	6	177.6	102.9	6	15.2%	-73.90 [-161.87, 14.07]
**Subtotal (95% CI)**			**27**			**27**	**46.5%**	**-106.89 [-150.78, -62.99]**
Heterogeneity: Tau² = 0.00; Chi² = 1.47, df = 2 (P = 0.48); I² = 0%								
Test for overall effect: Z = 4.77 (P < 0.00001)								
**5.1.2 MBC-Dopaminergic agents were left unregulated**								
Finazzi-Agrò 2003	182	125.4	5	332	76.3	5	10.3%	-150.00 [-278.66, -21.34]
Sartori 2022	377.6	169.3	39	386.4	163.4	39	17.3%	-8.80 [-82.65, 65.05]
Zong 2019	271.4	46	220	288.1	48.3	220	25.8%	-16.70 [-25.51, -7.89]
**Subtotal (95% CI)**			**264**			**264**	**53.5%**	**-31.79 [-83.97, 20.40]**
Heterogeneity: Tau² = 1167.95; Chi² = 4.15, df = 2 (P = 0.13); I² = 52%								
Test for overall effect: Z = 1.19 (P = 0.23)								
**Total (95% CI)**			**291**			**291**	**100.0%**	**-66.10 [-119.37, -12.82]**
Heterogeneity: Tau² = 2839.55; Chi² = 21.05, df = 5 (P = 0.0008); I² = 76%								
Test for overall effect: Z = 2.43 (P = 0.02)								
Test for subgroup differences: Chi² = 4.66, df = 1 (P = 0.03), I² = 78.5%								

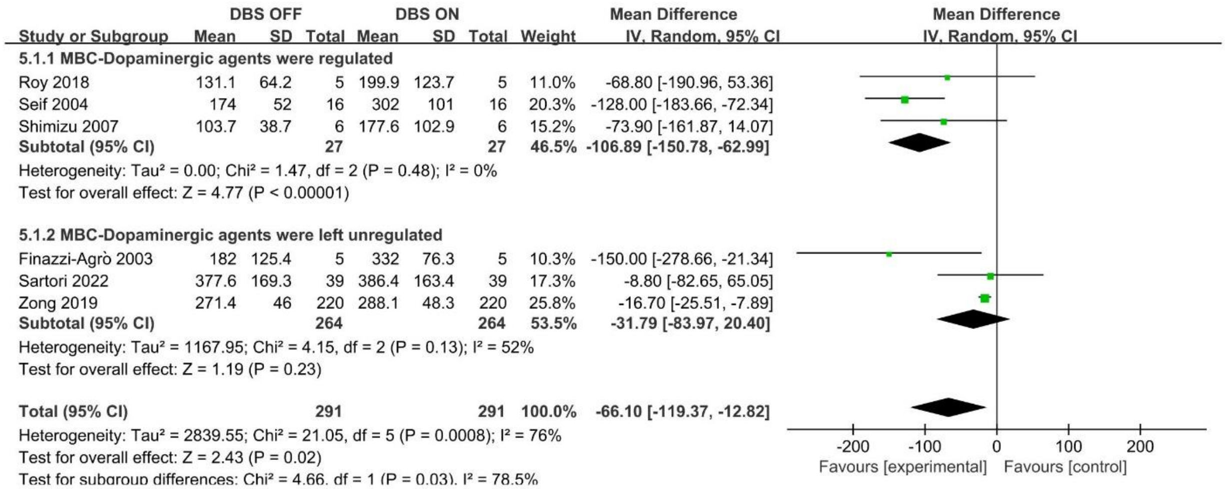

Supplementary Figure 5. Subgroup analysis of the impact of dopaminergic agents on Maximum bladder capacity (MBC).
